# A methodology for generating a tailored implementation blueprint: an exemplar from a youth residential setting

**DOI:** 10.1186/s13012-018-0761-6

**Published:** 2018-05-16

**Authors:** Cara C. Lewis, Kelli Scott, Brigid R. Marriott

**Affiliations:** 1 Kaiser Permanente Washington Health Research Institute, 1730 Minor Ave, Suite 1600, Seattle, WA 98101 USA; 2 Department of Psychological and Brain Sciences, Indiana University, 1101 E. 10th St, Bloomington, IN 47405 USA; 3 Department of Psychiatry and Behavioral Sciences, Harborview Medical Center, School of Medicine, University of Washington, Box 359911, 325 9th Ave, Seattle, WA 98104 USA; 4 Department of Psychological Sciences, University of Missouri, 315 Psychology Building, Columbia, MO 65211 USA

**Keywords:** Tailored implementation, Conjoint analysis, Mixed methods, Community partnership, Youth residential setting

## Abstract

**Background:**

Tailored implementation approaches are touted as more likely to support the integration of evidence-based practices. However, to our knowledge, few methodologies for tailoring implementations exist. This manuscript will apply a model-driven, mixed methods approach to a needs assessment to identify the determinants of practice, and pilot a modified conjoint analysis method to generate an implementation blueprint using a case example of a cognitive behavioral therapy (CBT) implementation in a youth residential center.

**Methods:**

Our proposed methodology contains five steps to address two goals: (1) identify the determinants of practice and (2) select and match implementation strategies to address the identified determinants (focusing on barriers). Participants in the case example included mental health therapists and operations staff in two programs of Wolverine Human Services. For step 1, the needs assessment, they completed surveys (clinician *N* = 10; operations staff *N* = 58; other *N* = 7) and participated in focus groups (clinician *N* = 15; operations staff *N* = 38) guided by the domains of the Framework for Diffusion [1]. For step 2, the research team conducted mixed methods analyses following the QUAN + QUAL structure for the purpose of convergence and expansion in a connecting process, revealing 76 unique barriers. Step 3 consisted of a modified conjoint analysis. For step 3a, agency administrators prioritized the identified barriers according to feasibility and importance. For step 3b, strategies were selected from a published compilation and rated for feasibility and likelihood of impacting CBT fidelity. For step 4, sociometric surveys informed implementation team member selection and a meeting was held to identify officers and clarify goals and responsibilities. For step 5, blueprints for each of pre-implementation, implementation, and sustainment phases were generated.

**Results:**

Forty-five unique strategies were prioritized across the 5 years and three phases representing all nine categories.

**Conclusions:**

Our novel methodology offers a relatively low burden collaborative approach to generating a plan for implementation that leverages advances in implementation science including measurement, models, strategy compilations, and methods from other fields.

**Electronic supplementary material:**

The online version of this article (10.1186/s13012-018-0761-6) contains supplementary material, which is available to authorized users.

## Background

Training and consultation are an empirically supported, integral set of strategies for implementing psychosocial interventions such as cognitive behavioral therapy (CBT). However, mounting literature suggests that training plus consultation, as a standalone implementation intervention, is unlikely to achieve full penetration and agency-wide long-term sustainment [2]. Complex settings, such as residential treatment, where team-based care is the primary mode of care delivery are likely to require additional strategies such as reorganization of clinical teams, revision of job descriptions, and engagement of clinical champions to fully integrate a new evidence-based psychosocial interventions (EBPs). With the demand for EBPs growing, it is important to determine how best to support their uptake, implementation, and sustainment to maximize the investment of community partners.

Tailoring strategies to the contextual factors that influence the implementation process is touted as critical to achieving success [3, 4]; however, few concrete examples of tailoring methods exist. Blueprints could arguably be tailored to the program level, site level, or organization level, depending on interest and resources. Sites within an organization can have significantly different contexts that may lead to unique tailored blueprints; however, it is unclear whether program level tailored blueprints are needed. Rather, tailoring strategies to the context has been recently described as the “black box” of implementation [5]. Revealing what is inside the black box is critical to advancing the science and practice of implementation and ensuring that successful implementation efforts are replicable.

A methodology for prospectively tailoring strategies has the potential to reveal a formal implementation blueprint, which Powell et al. [6] define as a plan that includes all goals and strategies, the scope of change, with a timeframe and milestones, as well as appropriate performance/progress measures. Two goals can be used to organize the steps in a tailoring methodology that yield a blueprint. The first goal is to identify determinants of practice, or the barriers and facilitators, of an organization. Krause et al. [7] recently compared brainstorming (open versus questionnaire guided) among health professionals, as well as interviews of patients and professionals, as methods for achieving this goal. They concluded that no single method emerged as superior and instead they recommend that a combined approach be used.

The second goal is to select and match implementation strategies to address the identified determinants (typically focusing on barriers) across the phases of pre-implementation (or exploration + preparation; [8]), implementation, and sustainment. Powell et al. [9] provide some methodological guidance in a recent conceptual paper for how to achieve this goal. Among their examples is conjoint analysis, which is described as having the advantages of providing a clear step-by-step method for selecting and tailoring strategies to unique settings that forces stakeholders to consider strategy-context match at a granular level. Importantly, Huntink et al. [10] found little to no difference in strategy generation across stakeholder categories (e.g., researchers, quality officers, health professionals) suggesting that involvement of stakeholders is important but equal representation and contribution is not necessary for sound strategy selection. Although literature is becoming available to aid in elucidating many of the critical steps to achieving these goals of determinant identification and strategy selection, no study has illustrated a method for establishing a formal tailored implementation blueprint.

The objective of this paper is to put forth a novel, empirically driven, collaborative methodology for generating a formal tailored blueprint using a CBT implementation at two sites of a youth residential setting as an exemplar. We will apply a model-driven, mixed methods approach to a “needs assessment” to identify the determinants of practice (focusing on barriers) and then pilot a modified conjoint analysis method to collaboratively generate an implementation blueprint. We will use a case study to describe this methodology in order to position others to engage in a structured, stakeholder-driven approach to prospectively build a tailored plan for implementation.

## Methods

### Formation of the academic-community partnership

Developing a tailored implementation blueprint would be challenging outside of an academic-community partnership. There are numerous models for how to initiate, operate, and sustain partnerships focused on EBP implementation (e.g., [11]). In our case example, administrators of a large Midwestern residential youth service treatment center, Wolverine Human Services, identified CBT as the optimal intervention to be implemented in conjunction with existing services after exploring available evidence-based practices on SAMHSA’s National Registry of Evidence-based Programs and Practices [12] and attending a series of workshops hosted by the Beck Institute. Based on typical implementation project timelines, we established a 5-year contract between the residential setting (key stakeholders included the Director of Clinical and Quality Services and the Vice President of Treatment Programs), the Beck Institute (purveyor), and implementation researchers to collaboratively pursue a 3-stage (i.e., pre-implementation, implementation, sustainment) process to integrate CBT into all levels of care provision. Drawing upon a community-based participatory research approach, all parties contributed to every phase described in detail below. Although this project was designed and funded for the purposes of achieving the clinical program change (i.e., CBT implementation), all parties were in support of contributing to the implementation science knowledge base. The Indiana University Institutional Review Board approved all study procedures.

### Participants

In the current case example, given the community partner’s implementation budget at the time the project was conceptualized, only two of six programs were selected for participation in the pilot endeavor. The two programs included a non-secure (unlocked, voluntary and mandated treatment) and secure (equipped with perimeter fencing, audio and video monitoring, locked, mandated treatment) program of Wolverine Human Services. Young people between the ages of 12 to 18 with a history of severe co-occurring mental health disorders and related behavioral/delinquency challenges (e.g., sexual, violent, and drug offenses) reside in the secure site, while the other site operates as a non-secure residential treatment program with youth in placement subsequent to truancy, and/or failure of other placements such as foster care. Typical mental health presenting problems include anxiety, mood disorders, oppositional defiant disorder and conduct disorders, reactive attachment and intermittent explosive disorder, bipolar, posttraumatic stress disorder, borderline personality disorder, with the majority struggling with externalizing problems, and comorbid disorders.

Participants contributing data to inform the tailored blueprint should represent all relevant stakeholders (e.g., clinical, operations, administrative). Participants in the case study consisted of staff stakeholders across levels at the two programs, including operations staff (i.e., youth care workers, shift/care coordinators), therapists, and leaders (e.g., managers, directors). The majority of staff (*N =* 76) shared their experience via surveys (therapists *n* = 10, operations staff *n* = 58, and other staff *n* = 7; one participant’s position was missing), and a subset of staff (*N =* 53) participated in one of seven focus groups (therapists *n* = 15, operations staff *n* = 38).

Therapists are typically the target of EBP implementation; at Wolverine Human Services, they are the highest ranking member of the treatment team and they are responsible for ensuring that the client’s needs are met by all staff. Therapists at Wolverine Human Services provide on-site education and training to the operation staff. Therapists provide family therapy/education at least monthly, and they conduct the initial and quarterly clinical assessments for each client. Therapists report directly to the court systems (juvenile justice or family/foster care) on the progress and needs of the youth. Therapists also provide individual and group therapy to the youth at the residential settings.

Operation staff is rarely the target of EBP implementation as they are not typically responsible for clinical care. However, Wolverine Human Services believed that to implement CBT with only therapists could limit or even undermine its impact. Operations staff is composed of youth care workers, who provide the majority of care and day-to-day youth oversight in the residential centers, and safety and support specialists, who coordinate staff and client assignments within programs of the organization as well as train staff and administer medications to youth.

### Procedures

**Fig. 1:**
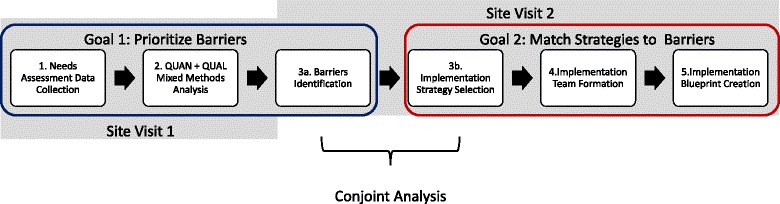
Tailoring methodology steps. This figure demonstrates the five steps of the tailoring methodology, grouped by goals and site visits

Our tailoring methodology consisted of five steps: (1) needs assessment data collection, (2) mixed methods analysis, (3a) determinant identification (focusing on barriers), (3b) implementation strategy selection, (4) implementation team formation, and (5) implementation blueprint creation; see Fig. 1. What follows are the methods and results from our case study applying these five steps to generate a tailored implementation blueprint for CBT implementation in Wolverine Human Services.

#### Step 1: needs assessment data collection

The needs assessment that leads to determinant identification can be conducted via numerous methods, each of which is likely to yield a different set and quantity of barriers and facilitators [7]. For this reason, mixed methods are encouraged. Determinant identification is likely to be optimized and streamlined if probing is guided by one of the 60+ models, frameworks, and theories for implementation [5, 15]. We selected the Framework for Dissemination to guide the quantitative and qualitative data collection in our case example because it was generated from the best available implementation research and an academic-community partnership that offered a relatively parsimonious set of six contextual domains for targeted analysis: (a) norms and attitudes (e.g., knowledge, expectations), (b) structure and process (e.g., mission, size), (c) resources (e.g., financial, physical, human, social, political capital), (d) policies and incentives (e.g., rewards or sanctions), (e) networks and linkages (e.g., conduits among stakeholders that enable information flow), and (f) media and change agents (e.g., active sources of information) [1]. As shown in the parenthetical examples, the first three domains capture the willingness and ability of key stakeholders to both implement and sustain new interventions, whereas the second three domains reflect sources of information and influence.

We administered quantitative measures assessing putative determinants of practice (e.g., attitudes towards evidence based practices, organizational climate, culture, leadership) mapping onto the Framework for Dissemination [1] to staff as part of the pre-implementation activities during the first site visit. The quantitative measures were selected from a database built by the first author through a National Institute of Mental Health funded systematic review of implementation measures [16]; the selected measures are listed and described in Table 1. Participants were excluded from this report only if they did not provide informed consent to allow data to be used for the purposes of research or if they had incomplete survey data on the majority of the measures. Participants generated a personal codename for their survey so that their responses could be anonymous but the research team could link responses over time. We calculated descriptive statistics (means, standard deviations, frequencies) for all applicable scale and total scores.

**Table 1:** Quantitative measures

Measure	Description
Employee Demographics Questionnaire (developed in-house)	This survey examined demographic information such as age, gender, ethnicity as well as theoretical orientation, training background, and clinical experience information (e.g., years of experience in counseling, previous training/experiences with CBT).
Evidence-Based Practice Attitudes Scale (EBPAS; Aarons, 2004)	The EBPAS is a 15-item measure that evaluates attitudes towards adopting evidence-based practices (EBPs) rated on a 5-point Likert scale of “Not at all” to “A very great extent.” This measure consists of a total score and four subscales, with a higher score indicating more positive attitudes towards the adoption of EBPs, (except for the divergence subscale in which a higher score indicates higher endorsed views of EBPs not being clinically useful). The four subscales are as follows: appeal (i.e., attitudes towards adopting if the EBP was intuitively appealing), requirements (i.e., attitudes towards adopting if it was required), openness (i.e., openness towards trying and using new interventions), and divergence (i.e., views of EBPs being unimportant and irrelevant to clinical experience) [21]. The EBPAS has displayed good internal consistency [21, 34] and construct and convergent validity [21, 34–36]. The internal consistency for EBPAS total score (Cronbach’s *α* = .88) was good for the sample as well as for the four subscales: requirements (Cronbach’s *α* = .91), appeal (Cronbach’s *α* = .94), openness (Cronbach’s *α* = .87), and divergence (Cronbach’s *α* = .76). Norm references include [37].
Attitudes Towards Standardized Assessment Scale (ASA; Jensen-Doss and Hawley 2010)	The ASA is a 22-item measure that assesses attitudes towards using standardized assessment on a 5-point Likert scale from “Strongly Disagree” to “Strongly Agree,” with a higher score indicative of more positive attitudes towards using standardized assessment in practice. The ASA is comprised of three subscales: Benefit over Clinical Judgment, Psychometric Quality, and Practicality. The ASA subscales have demonstrated good internal consistency and been found to be predictive of actual standardized assessment tool use [38]. The internal consistency of our sample was acceptable for Benefit over Clinical Judgment scale (Cronbach’s *α* = .74) and Psychometric Quality scale (Cronbach’s *α* = .68), but poor for the Practicality scale (Cronbach’s *α* = .58). Norm references include [38].
Organizational Culture Survey (OCS; Glaser 1983; Glaser et al. 1987)	The OCS measures the culture, or the normative beliefs and shared expectations of behavior [39, 40], of an organization. It is 31 items and uses a five-point Likert scale ranging from “To a Very Little Extent” to “To a Very Great Extent.” Higher scores demonstrate a more positive perception of the different aspects of the organization’s culture. The OCS is reliable, has high internal consistency, and contains six subscales: Teamwork, Morale, Supervision, Involvement, Information Flow, and Meetings [39]. The current sample’s internal consistency was good to excellent (Teamwork (Cronbach’s *α* = .93), Morale (Cronbach’s *α* = .94), Information Flow (Cronbach’s *α* = .85), Involvement (Cronbach’s *α* = .89), Supervision (Cronbach’s *α* = .94), and Meetings (Cronbach’s *α* = .93)). Norm references include [41].
Survey of Organizational Functioning (TCU SOF; Broome et al. 2007)	The SOF is 162-item measures that assesses the motivation factors, resources, staff attributes, climate, job attitudes, and workplace practices of an organization. The SOF includes the 23 scales of the Organizational Readiness for Change [42] plus nine additional scales examining workplace practices and job attitudes. All of the scales use a 5-point Likert scale of “Disagree Strongly” to “Agree Strongly” except for the Training Exposure scale, which uses a 5-point Likert scale of “None” to “4 or More,” and the Training Utilization-Individual Level and Training Utilization-Program-level scales, which utilize a 5-point Likert scale ranging from “Never” to “Almost Always.” Each scale score ranges from 10 to 50. All of the scales have shown acceptable psychometrics [43]. The internal consistency for most of the scales in the current study ranged from acceptable to excellent, with the scales Pressures for Change (Cronbach’s *α* = .58), Computer Access (Cronbach’s *α* = .33), Efficacy (Cronbach’s *α* = .55), Adaptability (Cronbach’s *α* = .50), Autonomy (Cronbach’s *α* = − .07), Stress (Cronbach’s *α* = .54), Change (Cronbach’s *α* = .48), Peer Collaboration (Cronbach’s *α* = .47), and Counselor Socialization (Cronbach’s *α* = .56) demonstrating poor to unacceptable internal consistency. Norm references include [42, 44–46]
Infrastructure Survey (Keough, Comtois, Lewis, and Landes 2013)	The Infrastructure Survey measures the impact of infrastructure (i.e., organization’s documentation, team format, performance evaluations/job descriptions, funding structure, staffing, and implementation outcomes) of clinical settings on the implementation and sustainment of empirically based treatments. The Infrastructure survey is comprised of 30 items and a recent exploratory factor analysis of this data [47] revealed four factors: Facilitative Staff Role, Flexibility, Adaptability, and Compatibility. The survey utilized a 5-point Likert scale ranging from “Strongly Disagree” to “Strongly Agree.” This study represents the first time this survey has been used since its creation, so its psychometrics have yet to be validated. The survey had good to excellent internal consistency for each scale (Facilitative Staff Role (Cronbach’s *α* = .92), Flexibility (Cronbach’s *α* = .89), Adaptability (Cronbach’s *α* = .93), and Compatibility (Cronbach’s *α* = .79). No norm references were available for the measure.
Opinion Leader Survey (Valente et al. 2007)	This survey elucidates the opinion leaders among an organization by requesting the respondent to name individuals “for whom you have relied on for advice on whether and how to use evidence-based practices for meeting the mental health needs of youth served by our agency” [48]. After listing these individuals, the respondent is asked to indicate how six additional criteria (i.e., sought advice from with respect to treatment, works for same agency as you, works for another agency or employer, is a subordinate or employees of yours, if your superior or supervisor, is your friend) apply to him/her.

Administrators nominated therapists and operations staff to participate in focus groups using purposeful sampling procedures highlighted by Palinkas and colleagues [13] to maximize the presence of both positive and negative viewpoints regarding CBT implementation. Each focus group included four to eight staff to allow all participants to share their viewpoints [17]. We conducted seven, 90-min focus groups (moderated by the first author; CCL) to elicit attitudes and beliefs from staff regarding the domains of the Framework for Dissemination [1]. In addition, focus groups discussed past implementation efforts, the effectiveness of implementation strategies used previously, the structure of usual care, and how CBT could fit into current practices. All focus groups were audio recorded, de-identified, and transcribed by a member of the research team.

We chose qualitative analysis procedures to evaluate the themes endorsed by focus group participants within the context of the Framework for Dissemination model domains [1]. We divided all focus group transcripts first into units of meaning to allow qualitative codes to represent specific thematic discussions of varying lengths. We then developed a coding dictionary based on the six domains identified in the Framework for Dissemination [1]; each of the six domains served as an overarching code for the focus group transcripts. Additional codes were added to the coding dictionary based on emergent topics identified in the focus group transcripts, which revealed one additional overarching code (or domain), communication. Fifty-six subcodes (i.e., codes to further clarify the overarching Framework for Dissemination domains) were also identified. Cross-cutting codes that spanned multiple overarching codes and subcodes included positive and negative valence statements, past, present, and future, desire for something (e.g., desire for training), and impact of a discussion topic on adolescents. Typical coder training and coding processes were enacted, the details of which can be found in Additional file 1.

#### Step 2: mixed methods analysis

Mixed methods analyses followed the QUAN + QUAL structure to serve the function of convergence and expansion in a connecting process [18], to first assess whether determinants emerged as barriers or facilitators based on quantitative norms (above or below, respectively) or frequent positive or negative endorsement in the focus group data (i.e., convergence), and second to gain additional insight into the nature of each barrier/facilitator (i.e., how does each barrier or facilitator manifest within sites) via the qualitative data (i.e., expansion). The quantitative data captured four of the six Framework for Dissemination (i.e., Norms & Attitudes, Structure & Process, and Networks & Linkages; Mendel et al. 2008) domains, while the qualitative data captured all six Framework for Dissemination domains. To generate the implementation blueprint, our team sought focused the determinant analysis to identify the barriers as they would require active strategies whereas we took note of facilitators and planned to leverage them wherever possible.

### Conjoint analysis

Conjoint analysis is a quantitative method wherein stakeholders evaluate and assign different values to product attributes, services, interventions, etc. [14]; this process helps clarify competing features or trade-offs to inform decisions about what should be of focus in the design of a product. Applied to the generation of a tailored implementation blueprint (i.e., a product), conjoint analysis can engage stakeholders in the clarification and prioritization of barriers and selection of strategies to enhance implementation. Stakeholder values can be revealed via various rating and sorting activities, ranging from consideration of feasibility and importance to assigning 100 priority points across available implementation strategies.

#### Step 3: conjoint analysis

##### 3a: barrier identification

In a second site visit, agency administrators rated the barriers identified from the mixed methods analysis on feasibility (i.e., high or low perceived ability to change the barrier) and on importance (i.e., high or low). To enhance the accessibility of this methodology, we wrote each of the 76 barriers on 3 × 5 note cards with either example items from the quantitative measures or code definitions from the qualitative data. We then presented to the administrators each notecard one at a time and asked them to place the barrier on a 2 × 2 poster board grid that had importance on the *Y*-axis and feasibility on the *X*-axis. Barriers that were assigned high feasibility and high importance ratings, such as staff CBT knowledge, were considered top priority.

##### Step 3b: implementation strategy selection

Potential implementation strategies were selected for the set of prioritized barriers in a matching process done collaboratively among researchers, administrators, and staff. Strategies were selected from the compilation generated by [6] in a review and synthesis including 68 unique strategies. Each strategy and its definition were presented to the stakeholders who rated strategy feasibility (i.e., high or low) given their budget and other resources. Subsequently, the CBT purveyor and the implementation researchers (who also had CBT expertise) rated the degree to which the strategy was integral to CBT adherence on a scale of 1 for low impact (i.e., not required for adherence), 2 for moderate impact (i.e., high priority for adherence), or 3 for high impact (i.e., a core element of CBT adherence [19]. Strategies were selected if they were rated as either high impact or high feasibility or both and then matched with one or more of the identified barriers based on the strategy’s potential mechanism of action.

#### Step 4: implementation team formation

Two implementation teams were formed, one for each program. These teams consisted of approximately 10 opinion leaders and champions representing all staff levels at each program (i.e., both therapists and operations staff). Members of the implementation team were selected based on a sociometric measure [20] (to identify opinion leaders) and the Evidence-Based Practice Attitudes Scale [21] (to identify champions) completed by therapists and operation staff during the needs assessment. Roles on the implementation team included Chair, Secretary, Program Evaluator, Incentives Officer, and Communication Officer (not all members held a formal role). These officer positions were chosen as they were the minimum set with clear responsibilities that would allow the team to get up and running and maintain itself over time; see Table 2. Implementation teams have a variety of functions including moving the project through the implementation, problem solving sustainability, and engaging stakeholders and increasing buy-in [22, 23]. In the case example, the main goals of the implementation teams were to oversee implementation strategy enactment and serve as in-house experts (both implementation & CBT).

**Table 2:** Officer positions and role responsibilities

Position	Role responsibility
Chair	Set the agenda and run the meetings
Secretary	Take minutes and ensure that meetings built from one another
Program Evaluator	Help collect and interpret data to track whether team activities have the intended influence
Incentives Officer	Ensure the team is being appropriately incentivized for this demanding, volunteer role (e.g., lunches during meetings, an additional PTO every 3 months)
Communications Officer	Strategically communicate out about the team’s activities to the rest of the organization

The CBT purveyor and implementation researchers met with the implementation teams from each of the two residential programs to provide an overview of the roles, functions, and responsibilities of the implementation teams. We asked teams to generate potential incentives for being members. Next, we presented the implementation teams with an overview of the prioritized barriers, specific examples that emerged from the needs assessment, and their implications for successful CBT implementation based on the latest research. We gave the implementation teams the opportunity to brainstorm additional implementation strategies that could be used to improve on or overcome the identified barriers. This step was important to ensure team members’ input, and language was incorporated into the final blueprint. Finally, the teams generated three top goals to guide their work during the pre-implementation period.

#### Step 5: implementation blueprint creation

We then organized the top-rated strategies into a 3-phase Implementation Blueprint guided by the implementation science literature and consensus among the CBT purveyor and implementation researchers regarding timing for optimal impact [9]. That is, we decided that it would not be feasible or wise to begin all strategies simultaneously but that some strategies (e.g., form implementation team) ought to be completed before others (e.g., training) are initiated. This implementation blueprint included key strategies that the team agreed would be implemented across pre-implementation, implementation, and sustainment phases, thereby providing a roadmap for the implementation effort. Final steps included a meeting between administrators, the CBT purveyor, and implementation researchers to review the Implementation Blueprint, discuss the roles and responsibilities of the implementation teams and consultants, and confirm the timeline.

## Results

Therapists were primarily female (*n* = 7, 70.0%), African American (*n* = 6, 60.0%) or Caucasian (*n* = 4, 40.0%), and had an average age of 35.7 (*SD* = 7.06). Most of the therapists had a Master’s degree (*n* = 9, 90.0%) and indicated Behavior Modification as their primary theoretical orientation (*n* = 7, 70.0%). Operation staff was predominantly males (*n* = 35, 60.3%), African Americans (*n* = 35, 60.3%) with a mean age of 36.7 (*SD* = 9.75). Similar to therapists, the majority of operations staff cited Behavior Modification as their primary theoretical orientation (*n* = 35, 60.3%). Operations staff had some college but no degree (*n* = 20, 34.5%) or a bachelor’s degree (*n* = 18, 31.0%).

The needs assessment (step 1) and mixed methods analysis (step 2) revealed 76 unique barriers. Exemplar barriers included climate and morale, communication, training, and teamwork and conflict. Three of the six Framework for Dissemination [1] domains captured by the quantitative measures revealed differences between Wolverine Human Services and national norms, suggesting these domains to be potential barriers or facilitators. In contrast, all six domains of the Framework for Dissemination were discussed as either barriers or facilitators in the qualitative data. See Additional file 2 for details regarding the barriers and facilitators, including quantitative results and exemplar quotes from staff. Conjoint analysis (steps 3a and 3b) prioritized (i.e., high feasibility and high importance) 23 of the 76 unique identified barriers, and 36 implementation strategies were selected (i.e., high impact or high feasibility or both) to address these barriers. Implementation teams were then formed to oversee implementation strategy enactment and serve as in-house experts (step 4). Finally, an implementation blueprint was compiled (step 5) in which the top-rated implementation strategies were organized across phases of implementation. Tables 3, 4, and 5 reflect the tailored blueprint for the pre-implementation, implementation, and sustainment phases, respectively, of the Wolverine Human Services CBT implementation project. As can be seen, 14 discrete strategies were selected for the pre-implementation phase (year 1), 19 for the implementation phase (years 2–4), and 12 for the sustainment phase (year 5). Using Waltz et al.’s [24] expert concept mapping solution for organizing strategies into conceptually distinct categories, pre-implementation included strategies across six of the nine categories: develop stakeholder relationships (*n* = 5, 35.7%), train and educate stakeholders (*n* = 3, 21.4%), support clinicians (*n* = 1, 7.1%), adapt and tailor to context (*n* = 1, 7.1%), use evaluative and iterative strategies (*n* = 1, 7.1%), and utilize financial strategies (*n* = 1, 7.1%). The implementation phase included strategies across eight of the nine categories: train and educate stakeholders (*n* = 5, 26.3%), support clinicians (*n* = 4, 21.0%), change infrastructure (*n =* 4, 21.0%), develop stakeholder relationships (*n* = 3, 15.8%), use evaluative and iterative strategies (*n* = 2, 10.5%), provide interactive assistance (*n =* 2, 10.5%), adapt and tailor to context (*n* = 1,5.3%), and utilize financial strategies (*n* = 1, 5.3%). Finally, the sustainment phase included strategies across six of the nine categories: train and educate stakeholders (*n* = 4, 33.3%), use evaluative and iterative strategies (*n* = 3, 25.0%), develop stakeholder relationships (*n* = 2, 16.7%), provide interactive assistance (*n =* 1, 8.3%), engage consumers (*n* = 1, 8.3%), and utilize financial strategies (*n* = 1, 8.3%).

**Table 3:** Blueprint for pre-implementation

Importance	Goal	Responsible	Feasibility	Impact	Implementation category	Action step
H	1, 2, 3	IT	H	3	Develop stakeholder interrelationships	Implementation team––reserve biweekly meetings
H	1, 3	IT	L	1.5	Support clinicians	Restructure clinical teams
H	3	B	H	2	Train and educate stakeholders	Select training methods that fit preferences of staff
H	1, 2, 3	IT	L	3	Develop stakeholder interrelationships	Recruit, designate, and train for leadership (pick chair/lead)
H	3	B/IT	L	3	Adapt and tailor to context	Fit intervention to clinical practice (link points and levels with CBT and outcome monitoring)
H	1, 3	B/IT			Use evaluative and iterative strategies	Develop and implement tools for quality monitoring (identify program level measures)
M	3	B	H	1	Develop stakeholder interrelationships	Develop implementation glossary
M	3	B	H	1	Develop stakeholder interrelationships	Develop structured referral sheets
L	3	B	L	2	Train and educate stakeholders	Prepare client-facing psychoeducational materials regarding mental health problems
M	1	IT	L	3	Utilize financial strategies	Shift resources for incentives, support and to reduce turnover
M	1, 2	B/IT	H	2	Develop stakeholder interrelationships	Conduct local consensus discussions––mix with educational meetings
L	1, 2	IT	H	1	Train and educate stakeholders	Conduct educational meetings
L	3	IT	L	2	Change infrastructure	Modify context to prompt new behaviors––change note template
M	3	B/IT	L	3	Utilize financial strategies	Access new funding

“Importance” contains “H” for “High” (i.e., strategy must be enacted because it targets a highly important barrier), “M” for “Moderate” (i.e., the strategy should be prioritized if resources are available), or an “L” for “Low” (i.e., strategy should only be enacted if time and resources are available). “Goal” contains a 1, 2, or 3 to indicate which of the top 3 goals the strategy would primarily target. “Responsible” reflects whether the strategy should be enacted by the “IT” (i.e., Implementation Team at Wolverine Human Services) or “B” (i.e., Beck Institute or other external experts). “Feasibility” contains either “H” for “High” or “L” for “Low” in terms of the ease with which a given strategy could be enacted. “Impact” contains a score from 1 to 3 to reflect the degree to which the strategy would likely impact the fidelity with which CBT would be delivered. “Implementation Category” is derived from an expert-engaged concept mapping exercise in which nine conceptually distinct categories emerged: adapt and tailor to the context, change infrastructure, develop stakeholder interrelationships, engage consumers, provide interactive assistance, support clinicians, train and educate stakeholders, utilize financial strategies, and use evaluative and iterative strategies. Implementation strategy contains the name of the strategy to be enacted. Timeline: Revisit in 6–8 months (truncated surveys, focus groups)

Note. Goals: *1* improve climate, satisfaction, communication, and teamwork; *2* re-establish consistency/quality of physical restraints; *3* Prep materials to support CBT

**Table 4:** Blueprint for implementation

Importance	Goal	Responsible	Feasibility	Impact	Implementation category	Action step
H	1, 2, 3	B	H	3	Train and educate stakeholders/provide interactive assistance	Beck/IU training/supervision
H	1, 2, 3	IT	L	2	Develop stakeholder interrelationships	Hold cross-staff clinical meetings
H	1, 3	B/IT	H	2	Adapt and tailor to context	Facilitate, structure, and promote adaptability (Beck to work with IT to modify CBT to fit the sites)
H	2	B	L	3	Train and educate stakeholders	Conduct educational outreach visits
H	3	IT	L	3	Utilize financial strategies	Shift resources (ensure strategy for monitoring outcomes)
H	2	IT	H	1	Develop stakeholder interrelationships	Identify early adopters (have person shadowed, talk in clinical meetings about overcoming barriers)
H	2	B	L	3	Provide interactive assistance	Provide clinical supervision––include IT on calls
H	1, 2	B/IT	L	3	Train and educate stakeholders	Use train-the-trainers strategies
H	2, 3	IT	L	3	Change infrastructure	Increase demand––present data to courts and state level
H	2	IT	H	2	Support clinicians	Change performance evaluations, change professional roles
M	2	B/IT	H	1	Use evaluative and iterative strategies	Develop and institute self-assessment of competency
M	2, 3	IT	H	2	Develop stakeholder interrelationships	Capture and share local knowledge
M	2	IT	H	1	Support clinicians	Remind clinicians
L	3	B/IT	L	2	Train and educate stakeholders	Prep CBT client handouts (Beck to provide examples)
L	1, 2	B/IT	L	2	Utilize financial strategies	Alter incentives (certification, vacation, salary)
L	1, 3	B/IT	L	2	Support clinicians	Facilitate relay of clinical data to providers (data parties)
L	1, 2	IT	L	2	Support clinicians	Modify context to prompt new behaviors
L	1, 2, 3	IT	L	2	Train and educate stakeholders	Shadow other experts
L	1, 2, 3	IT	L	2	Use evaluative and iterative strategies	Obtain and use consumer and family feedback (exit interviews and surveys)

“Importance” contains “H” for “High” (i.e., strategy must be enacted because it targets a highly important barrier), “M” for “Moderate” (i.e., the strategy should be prioritized if resources are available), or an “L” for “Low” (i.e., strategy should only be enacted if time and resources are available). “Goal” contains a 1, 2, or 3 to indicate which of the top 3 goals the strategy would primarily target. “Responsible” reflects whether the strategy should be enacted by the “IT” (i.e., Implementation Team at Wolverine Human Services) or “B” (i.e., Beck Institute or other external experts). “Feasibility” contains either “H” for “High” or “L” for “Low” in terms of the ease with which a given strategy could be enacted. “Impact” contains a score from 1 to 3 to reflect the degree to which the strategy would likely impact the fidelity with which CBT would be delivered. “Implementation Category” is derived from an expert-engaged concept mapping exercise in which nine conceptually distinct categories emerged: adapt and tailor to the context, change infrastructure, develop stakeholder interrelationships, engage consumers, provide interactive assistance, support clinicians, train and educate stakeholders, utilize financial strategies, and use evaluative and iterative strategies. Implementation strategy contains the name of the strategy to be enacted. Timeline: 3 years total; 3–5 day training every 6 months

Note. Goals: *1* continue to enhance climate, teamwork, communication, attitudes, and satisfaction; *2* increase CBT knowledge, skill––integrate into care; *3* demonstrate benefit to youth

**Table 5:** Blueprint for sustainment

Importance	Goal	Responsible	Feasibility	Impact	Implementation category	Action step
H	1, 2, 3	IT	H	3	Develop stakeholder interrelationships	Engage implementation team
H	1, 3	IT	L	2	Develop stakeholder interrelationships	Hold cross-staff clinical meetings
H	3	IT	L	3	Use evaluative and iterative strategies	Develop and implement for quality monitoring––must monitor fidelity through observation regularly and randomly
H	1, 3	IT	H	1	Train and educate stakeholders	Conduct educational meetings––hold regularly for new staff and as refreshers
H	1, 3	IT	L	3	Train and educate stakeholders	Use train-the-trainer strategies––only those certified in CBT
H	1, 2, 3	IT	L	2	Provide interactive assistance	Centralize technical assistance––create standard operating procedure for training and use of CBT at each staff level
L	1, 2	IT	L	2	Utilize financial strategies	Alter incentives––provide raise earlier based on competency
L	1, 3	IT	L	2	Use evaluative and iterative strategies	Obtain and use consumer feedback w/PQI data collection
L	1, 3	IT	L	2	Train and educate stakeholders	Shadow other experts––elongate period for new staff
L	1, 2, 3	IT	L	2	Train and educate stakeholders	Develop learning collaborative
L	3	B/IT	L	2	Use evaluative and iterative strategies	Stage implementation scale-up to generate plan across site
L	3	B/IT	L	2	Engage consumers	Use mass media––get press release out with data from implementation

“Importance” contains “H” for “High” (i.e., strategy must be enacted because it targets a highly important barrier), “M” for “Moderate” (i.e., the strategy should be prioritized if resources are available), or a “L” for “Low” (i.e., strategy should only be enacted if time and resources are available). “Goal” contains a 1, 2, or 3 to indicate which of the top 3 goals the strategy would primarily target. “Responsible” reflects whether the strategy should be enacted by the “IT” (i.e., Implementation Team at Wolverine Human Services) or “B” (i.e., Beck Institute or other external experts). “Feasibility” contains either “H” for “High” or “L” for “Low” in terms of the ease with which a given strategy could be enacted. “Impact” contains a score from 1 to 3 to reflect the degree to which the strategy would likely impact the fidelity with which CBT would be delivered. “Implementation Category” is derived from an expert-engaged concept mapping exercise in which nine conceptually distinct categories emerged: Adapt and Tailor to the Context, Change Infrastructure, Develop Stakeholder Interrelationships, Engage Consumers, Provide Interactive Assistance, Support Clinicians, Train and Educate Stakeholders, Utilize Financial Strategies, Use Evaluative and Iterative Strategies. Implementation Strategy contains the name of the strategy to be enacted. Timeline: Monitor 1 year post formal training

Note. Goals: *1* train new staff efficiently; *2* maintain climate and communication; *3* sustain integration and penetration of CBT

## Discussion

This study put forth a collaborative, model-based, mixed qualitative- and quantitative-informed methodology for developing a blueprint to guide the work of implementation teams across pre-implementation, implementation, and sustainment. We applied the methodology in the context of a youth residential center that sought to implement cognitive behavior therapy (CBT). Seventy-six unique barriers to implementation emerged from a mixed methods needs assessment guided by the Framework for Dissemination [1]. A modified, low technology conjoint analysis revealed 23 barriers that agency administrators perceived to be of high importance and highly feasible to address (e.g., teamwork, training, morale, communication). After forming implementation teams consisting of influential staff representatives per social network data, 36 strategies were matched to the prioritized barriers based on their feasibility and potential impact on implementing CBT with fidelity. The strategies were mapped across the phases of pre-implementation (e.g., develop implementation glossary, conduct local consensus discussions, modify context to prompt new behaviors), implementation (e.g., conduct educational outreach visits, remind clinicians, shadow experts), and sustainment (e.g., use train-the-trainer strategies, revise job descriptions) and assigned to either (or both) the implementation teams or purveyors for completion. The resulting blueprint highlights overarching goals of each phase, priorities (high versus low), a timeline, and concrete strategies agreed upon by all stakeholders, the purveyor, and researchers to serve as a roadmap to guide the collaborative effort. This methodology leverages the best available research, clarifies the subsequent process and roles for stakeholders, allows for cost mapping of high (e.g., regularly engage implementation teams) versus low (e.g., access new funding) priority implementation strategies, and ensures strategies target the unique contextual barriers of a site.

With respect to the first goal of this methodology—i.e., prioritize barriers––it remains an empirical question as to how different the identified barriers would have been if another model had guided the quantitative and qualitative evaluation. That is, although the Framework for Dissemination [1] was selected as the model to guide the needs assessment in this case example, this methodology could be employed with a variety of different frameworks such as the Consolidated Framework for Implementation Research (CFIR; [25], the Exploration Preparation Implementation Sustainment (EPIS; [8]) model, or the Dynamic Sustainability Framework [26]. Indeed, there are over 60 dissemination and implementation frameworks, theories, and models in use, each with unique and overlapping constructs identified as potential contextual barriers to the implementation process [15], which will influence measurement and potentially the barriers identified. For instance, the Framework for Dissemination acknowledges the importance of opinion leaders but does not emphasize broader leadership impacts whereas the EPIS model explicitly highlights both senior leadership buy-in and team-level leadership in the needs assessment phase. Fortunately, there are ongoing efforts to map quantitative measures to the CFIR, which is the most comprehensive taxonomy of constructs, and provide information on the degree to which these measures are pragmatic (e.g., low cost, brief, actionable) and psychometrically strong [16]. There is also an evolving website that lists common constructs, associated measures, and information on the models identified by Tabak and colleagues in their review [27]. Both of these emerging resources will prove useful for identifying barriers in future research. Moreover, use of frameworks will help to focus the assessment on key constructs that yield a manageable set of barriers that can otherwise quickly become unwieldy (e.g., over 600 determinants) if open brainstorming is used as the method [7]. Until we know more about robust contextual predictors or mediators of implementation, comprehensive assessments of this nature will likely need to be conducted.

With respect to the second goal of this methodology—i.e., match strategies to barriers—there are numerous structured processes emerging (e.g., conjoint analysis, intervention mapping, group model building) that could be applied [5]; however, all require some knowledge of available implementation strategies. Powell et al. [6, 9] offer a compilation of discrete implementation strategies that can serve as a menu from which to select options. In the current methodology, stakeholder perception of strategy feasibility and purveyor expertise regarding strategy impact on CBT fidelity guided the selection process and implementation experts refined the matching to barriers and staging across implementation phases. There is little empirical guidance regarding which strategies target which barriers, how strategies act to create change (i.e., mechanisms), and at which point in the implementation process a strategy may be most potent. Recent work on “Expert Recommendations for Implementing Change” endeavors to address some of these gaps in the literature [19]. They recently produced a taxonomy of implementation strategies, a conceptual map of strategies across nine categories [24] and results from an expert-led hypothetical matching exercise. Results are forthcoming for their expert-led mapping of strategies to implementation phase for their hypothetical scenarios. We applied the results of their work to date in the current methodology to summarize the blueprint in implementation strategy terms and found that although all nine categories were included across one of the three phases, some were observed in high frequency across phases (e.g., develop stakeholder interrelationships, train, and education stakeholders) whereas others emerged only once (e.g., engage consumers). We do not yet know if this distribution was optimal or if the strategies selected effectively targeted the key determinants in these programs. In a recent publication, Boyd and colleagues found that certain strategy categories (i.e., financial strategies) were significantly related to implementation outcomes whereas others were not [28]. More research is needed to enhance our proposed methodology with empirical guidance for strategy selection and matching.

We made significant modifications to the traditional conjoint analysis process in order to lower the cost and increase accessibility. For instance, we opted for note cards and poster board versus Sawtooth Software packages [29] to prioritize barriers. Although this may have compromised the rigor of the method, there is concern that a divide is growing between best practices from implementation science and what is actually happening (and feasible) in the practice world [16, 30]. For example, concept mapping is gaining empirical support as an effective method for engaging stakeholders in systematically prioritizing barriers [31]. However, concept mapping is resource intensive (e.g., electronic devices are needed for survey completion, special software needed for data collection and analysis) and requires content and methodological expertise (i.e., to develop the content for the software, analyze, and interpret the data). Also, access to the internet is not ubiquitous especially in rural communities such as the youth residential center in which our case example occurred. Moreover, although it is relatively more feasible than concept mapping, the proposed methodology has associated personnel burden insofar as the quantitative surveys require some level of analysis (e.g., descriptive statistics) and possible comparison (e.g., calculation of effect size in comparison to norms or to a neutral midpoint) and the qualitative data require skilled personnel for formally coding, as well as the cost of transcriptions. With respect to the quantitative assessment component, there is a relatively new effort to develop “pragmatic measures” that could be used independently by stakeholders to inform an implementation effort [16]. With respect to insights gained in a more qualitative manner, it may be that rapid ethnography is a viable alternative [32]. Regardless, tailoring strategies to the context is inherently more work than a standardized approach.

There are several differences between our work and that of the Tailored Implementation for Chronic Disease (TICD) [10] including the target disease/disorder (behavioral health versus chronic health conditions), setting (small, rural residential care center versus large healthcare organizations), participants (providers and staff with little education beyond high school versus doctorate level professionals), and country in which the studies were conducted (USA versus five European countries). There were also differences in our methodologies, including that we presented stakeholders with implementation strategies (based on a published compilation [6]) that they then rated whereas they encouraged open brainstorming followed by a structured interview that brought evidence from implementation science to bear. Despite these differences, one similarity in our methodologies is that barriers were only assessed and prioritized once at the beginning of the process to inform the tailored approach to implementation. It may be the case that an approach more aligned with continuous quality improvement would lead to better results, although this would also demand more resources. In our current methodology, we do reassess contextual factors every six to 12 months to determine if any barriers have re-emerged.

There are several noteworthy limitations to the current study. First, the methodology was piloted in a youth residential center, the results of which may have limited generalizability to other settings. Second, we did not work with the two programs (secure and non-secure) of the residential center separately to see if different blueprints would have emerged because it was the preference of the administrators to keep some level of continuity/standardization in the project despite acknowledgement that different barriers emerged between the programs. Third, the blueprints yielded a rich and complex suite of implementation strategies for enactment across the phases of implementation (i.e., 45 unique strategies were selected across nine categories). If the goal had been to generate the most parsimonious approach, it is unclear which strategies were necessary to address which determinants and when. Fourth, the blueprints focused on barriers and did not include facilitators despite that our needs assessment contained this type of information. It is an empirical question whether targeting barriers is more or less efficient than a combined approach (target barriers and leverage facilitators). Fifth, although our preliminary results suggest that the enactment of the pre-implementation blueprint was very successful in ameliorating the prioritized barriers [33], we are unable to report on the effectiveness of the strategies across the implementation and sustainment phases because the project is ongoing and no comparison conditions exist. Finally, this approach necessitates prospective tailoring, which assumes the context is static, a fallacy that is highlighted in the Dynamic Sustainability Framework [26]. Empirical work is needed to separate out the differential impacts of one-time prospective tailoring versus iterative adaptations over time. In our annual reassessment of Wolverine Human Services, we have observed that some barriers re-emerge and need to be addressed and that new implementation strategies are prioritized that we did not plan for in our initial blueprint.

## Conclusions

Despite the apparent need to select and match strategies to fit the implementation context, few methodologies exist. In this paper, we presented a model-based, mixed qualitative, and quantitative methodology for generating a tailored blueprint by achieving two goals: (1) prioritizing barriers and (2) matching strategies to prioritized barriers. Our methodology is being applied to an implementation of CBT in youth residential settings and this process yielded a manageable set of prioritized barriers and a clear plan for which strategies to engage by whom and when in the process via an implementation blueprint. Collaboratively developed blueprints from a methodology of this nature offer numerous benefits, notably around transparency and stakeholder ownership of the process. We will examine the effectiveness of the resulting blueprint on implementation outcomes as the process unfolds using an interrupted time series design. However, research is needed to determine whether this methodology outperforms other approaches.

## Additional file

Additional file 1: Details regarding the qualitative coder training and coding process. This file provides additional information regarding the training of qualitative coders and the process followed for coding qualitative data. (DOCX 14 kb)

Additional file 2: Barriers and facilitators. This file includes details regarding the barriers and facilitators, including quantitative results and exemplar quotes from staff. (DOCX 19 kb)

## Abbreviations

CBT: Cognitive behavioral therapy
CFIR: Consolidated Framework for Implementation Research
EBPs: Evidence-based psychosocial interventions
EPIS: Exploration Preparation Implementation Sustainment
