# Supplementary material for: A methodology for generating a tailored implementation blueprint: an exemplar from a youth residential setting
Source: Implement Sci. 2018 May 16;13:68. doi: 10.1186/s13012-018-0761-6 (PMC5956960; doi:10.1186/s13012-018-0761-6)
Supplement: Supplementary file 1 — Details regarding the qualitative coder training and coding process. This file provides additional information regarding the training of qualitative coders and the process followed for coding qualitative data. (DOCX 14 kb) [file 13012_2018_761_MOESM1_ESM.docx]

**Additional File 1: Details Regarding the Qualitative Coder Training and Coding Process**

All overarching and subthemes were organized into thematic hierarchies and the second author (KS) worked with the first author (CCL) and the trained coders to establish definitions and example units of meaning for all overarching, sub, and cross cutting themes. To promote reliable coding of the focus group transcripts, three coders were trained to competency on the coding dictionary definitions and examples by the second author (KS). The three coders participated in the development of the coding dictionary and then participated in two, three-hour coding training meetings structured such that the coders were able to familiarize themselves with the coding dictionary, practice assigning codes to units of meaning in the transcripts, and ask questions about the definitions of codes.

Upon completion of coder training, the three coders completed a pilot test of the coding dictionary to test the dictionary for theme saturation and to evaluate trainee inter-rater reliability. The coders employed the coding dictionary to assign codes to units of meaning within one focus group transcript using QSR NVIVO 10 software. During the pilot test procedure, the coders integrated the coding dictionary into the NVIVO software by creating individual nodes and subnodes for each of the overarching themes, subthemes, and cross cutting themes identified. Throughout pilot coding, additional weekly meetings were held with the first and second authors to ensure that coding was being completed correctly, to address any themes not accounted for by the coding dictionary, and to ensure that coders maintained inter-rater reliability. Upon completion of the pilot coding procedure, the coding hierarchy and dictionary were revised and formal definitions were established for each of the themes and subthemes.

Formal coding procedures were completed on all seven focus group transcripts using NVIVO 10 qualitative analysis software. Two trained coders independently completed coding for each of the transcripts using a blocked design to promote efficiency of coding while maintaining the ability to assess inter-rater reliability (i.e. two out of three available coders coded each transcript). Weekly, hour-long meetings were held to ensure ongoing inter-rater reliability and to establish consensus across coders for each of the seven transcripts.
