# Supplementary material for: A methodology for generating a tailored implementation blueprint: an exemplar from a youth residential setting
Source: Implement Sci. 2018 May 16;13:68. doi: 10.1186/s13012-018-0761-6 (PMC5956960; doi:10.1186/s13012-018-0761-6)
Supplement: Supplementary file 2 — Barriers and facilitators. This file includes details regarding the barriers and facilitators, including quantitative results and exemplar quotes from staff. (DOCX 19 kb) [file 13012_2018_761_MOESM2_ESM.docx]

**Barriers and Facilitators**

**Norms and attitudes.** Climate and morale (*t*(310) = 5.15, *p* < 0.01) and attitudes about the benefit of standardized assessment over clinical judgment (*t*(1475) = 1.97, *p* < 0.05) emerged as potential barriers for the residential center. The qualitative data aligned with and expanded upon these findings, as morale issues were highlighted as key barriers to implementation by both therapists and operations staff. Therapists discussed that they are “…smack dab in one of those, do I want to do this anymore? Do I want to be here anymore modes, to be honest” and that “eagerness and excitement gets less as you go from top executive down.” Operations staff indicated that:

*“…but the morale like I said, be there for your coordinators, you know. Instead of being put down all the time, showing them what’s going wrong all the time. Like I tell the kids, you know, instead of putting somebody down today, lift somebody up you know it feels good, you know what I’m saying? …”*

Therapists also noted difficulties in building relationship with youth, as:

*“…you get to spend a couple months just building the rapport and so sometimes it’s hard for [the adolescents] and I’ve seen a big decrease in like the support that they feel. They may have their worker come up once a month for 10 minutes, writing stuff down, you know and they may leave and they get a 10 minute phone call, so I’ve seen a big decrease in the support that, I don’t know what the answer to that is.”*

**Structure and process.** Issues related to training (*t*(526) = 5.65, *p* < 0.01) also emerged as potential barriers to implementation. Therapists and operations staff shared similar viewpoints regarding their desire for high quality training, lack of current training options, and specific need for training strategies for enhancing the care provided to the adolescents. Both therapists and operations staff had generally negative views about the lack of training options and endorsed a desire for additional training in strategies for dealing with youth, interventions for mental health, techniques for tailoring intervention to male and female adolescents, and strategies for discussing trauma with the adolescents. Therapists specifically indicated that:

*“…and I’ve heard a lot of them talk about if we only had more training, now I don’t know what they mean by that word. Um, and maybe because we’re only as good as our staff that are in the unit right now, so I don’t know maybe if there was more like training not as in like the organization of the unit, and when is paperwork due, and when are home visits, just actually like what can we do for these kids.”*

Operations staff indicated that:

*“I mean, I would, I would like to have training on something like [clears throat]. On how to talk to ‘em about real emotional situations they’re tellin’ me about,” and, “I want real training like serious training on how to meet these issues as they present themselves.”*

**Networks and linkages.** Teamwork and conflict (*t*(310) = 4.34, *p* < 0.01) also emerged as a potential barrier to implementation. Regarding teamwork, therapists reported that:

*“It’s simple things. Like just talking with each other and you know, having being able to like for example we used to be able to take breaks together because there’d be two coordinators. So, you know, we’re both on break let’s hang out. Let’s go, you know, we got 15 minutes let’s go.”*

Several Framework of Dissemination domains (i.e., Resources, Policies and Incentives, and Media and Change agents) either were not evaluated in the quantitative data or did not emerge as significant barriers or facilitators. However, all three of these domains were captured by the qualitative data. Resources were highlighted as a barrier, with lack of technology and funding issues emerging among therapists and operations staff such as, “What’s necessary to happen is funding that we do not receive for after-care and home services,” and, “We don’t have computers and internet to do our work.”

Policies and incentives related to staff roles and purpose of paperwork workload also emerged as implementation barriers. Regarding roles, therapists discussed that “… being here 7 months, and I just like…just last week I just said I can’t do this anymore. I’m supposed to be here for therapy. I’m spending most of my time doing reports and then it’s just like on a whim trying to see a kid.” Operations staff echoed these concerns about roles, stating that:

*“Well I see my job description as being: oversee the site, make sure the kids are safe and the site is safe and that entails a whole lot,” and “then there’s something when something pops off or you know, there’s a problem, you’re now the therapist, you are the team leader. You are sometimes the parent.”*

Therapists and operations staff were also generally negative about the purpose of the paperwork required by the organization. Therapists discussed that:

*“Weeklys [reports], on a kid, and you have to change it up, on a kid that has been there and has been doing pretty well for weeks…and then they want you, they say well this is the same from last week, well he was the same from last week you know, but they want you to change it up and I’m like what more can I say that he [the adolescent] continues to participate in the individual [therapy], continues to participate in the group [therapy], what else?”*

Operations staff also expressed negative views of current paperwork requirements, stating that, “I mean like I’ve been coordinating for 10 years…they come up with like 50 new forms that has nothing to do with nothing, it’s just a waste of time.”

Media and change agents emerged as both barriers and facilitators of implementation in the qualitative data. Some therapists and operations staff noted that, “Ya know, we need people to come in that know what they’re doing, what they’re talking about,” while others highlighted concerns about trainers’ ability to relate, “You know what I would like? See, I would like to see people that do the trainings come in and try to do what we do,”

Finally, the theme of “Communication” emerged as a domain not captured by the Framework for Dissemination, and the quantitative data found this theme to be statistically significant (*t*(527) = 4.76, *p* < .01), with the residential treatment center having more negative perceptions of communication than national norms. Qualitative data also identified communication as a significant implementation barrier, as communication issues were the most frequently endorsed difficulties among both therapists and operations staff. Therapists discussed difficulties in communication in meetings stating that “…because there now are not like the 1998 meetings that he had, it’s kind of like we never wanna go just because we have other stuff we have to do.” Therapists also indicated that “…a barrier of communication, especially for myself, is, we have supervisor people in different locations, you’re not seeing them, so it’s through telephone, email, it’s not as effective as face-to-face communication.”

However, therapists and staff also endorsed positive views about communication, suggesting evidence that communication may also serve as a facilitator. For example, operations staff stated that “I think, totally, I think the big picture, we’re on the same page [in terms of communication], I do,” and “…I think we all tend to share information very well. Um, whether we’re forced to, or whether we just do it because we know that’s how it needs to be done.”

Overall, both the quantitative and qualitative data identified several barriers and facilitators to CBT implementation across the two sites. Although a number of Framework for Dissemination domains are highlighted above as implementation barriers, several topics also served as possible facilitators of successful implementation. Specifically, the quantitative and qualitative data both highlighted themes related to norms and attitudes as both barriers and facilitators, and structure and process and networks and linkages as solely barriers to implementation. The quantitative data suggested that resources were neither a barrier nor facilitator, while the qualitative data identified a number of resource related barriers. The qualitative data identified policies and incentives as a barrier and media and change agents as both barriers and facilitators, while quantitative measures were unable to capture these domains given a lack of validated measures to assess these constructs. Finally, communication was identified as a barrier in the quantitative data and both a barrier and facilitator in the qualitative data. While several of the domains demonstrated convergence between the quantitative and qualitative data collection methods, these findings highlight the unique data that can be gleaned by using a mixed methods approach. The use of qualitative methods enabled the identification of barriers not captured by the quantitative data, and highlighted facilitators that may be especially important to leverage for successful implementation of CBT.
